# A Dual-Function “TRE-Lox” System for Genetic Deletion or Reversible, Titratable, and Near-Complete Downregulation of Cathepsin D

**DOI:** 10.3390/ijms24076745

**Published:** 2023-04-04

**Authors:** Heather M. Terron, Derek S. Maranan, Luke A. Burgard, Frank M. LaFerla, Shelley Lane, Malcolm A. Leissring

**Affiliations:** 1 Institute for Memory Impairments and Neurological Disorders, University of California, Irvine, Irvine, CA 92697, USA; 2 Department of Neurobiology and Behavior, University of California, Irvine, Irvine, CA 92697, USA

**Keywords:** Alzheimer disease, cathepsin D, Cre-Lox technology, CRISPR, gene regulation, tetracycline regulatory element

## Abstract

Commonly employed methods for reversibly disrupting gene expression, such as those based on RNAi or CRISPRi, are rarely capable of achieving >80–90% downregulation, making them unsuitable for targeting genes that require more complete disruption to elicit a phenotype. Genetic deletion, on the other hand, while enabling complete disruption of target genes, often produces undesirable irreversible consequences such as cytotoxicity or cell death. Here we describe the design, development, and detailed characterization of a dual-function “TRE-Lox” system for effecting either (a) doxycycline (Dox)-mediated downregulation or (b) genetic deletion of a target gene—the lysosomal aspartyl protease cathepsin D (CatD)—based on targeted insertion of a tetracycline-response element (TRE) and two LoxP sites into the 5′ end of the endogenous CatD gene (*CTSD*). Using an optimized reverse-tetracycline transrepressor (rtTR) variant fused with the Krüppel-associated box (KRAB) domain, we show that CatD expression can be disrupted by as much as 98% in mouse embryonic fibroblasts (MEFs). This system is highly sensitive to Dox (IC_50_ = 1.46 ng/mL) and results in rapid (t_1/2_ = 0.57 d) and titratable downregulation of CatD. Notably, even near-total disruption of CatD expression was completely reversed by withdrawal of Dox. As expected, transient expression of Cre recombinase results in complete deletion of the *CTSD* gene. The dual functionality of this novel system will facilitate future studies of the involvement of CatD in various diseases, particularly those attributable to partial loss of CatD function. In addition, the TRE-Lox approach should be applicable to the regulation of other target genes requiring more complete disruption than can be achieved by traditional methods.

## 1. Introduction

Revolutionary advances in molecular biology have made it possible to readily manipulate gene expression via a variety of approaches, including Cre-Lox technology [1,2], inducible Tet-On/Off systems [3,4], RNA interference (RNAi) [5,6], and CRISPR/Cas-based approaches [7,8,9,10]. Methods that permanently modify genomic DNA (e.g., Cre-Lox, CRISPR/Cas) generally produce the most definitive effects, such as complete knock-out (KO) of gene expression, but can be time-consuming and expensive to implement. Moreover, for many applications complete deletion of a gene is not desirable. Methods that indirectly modify gene expression (e.g., antisense oligonucleotides, RNAi, CRISPRi) tend to be comparatively faster and less expensive, but typically require extensive optimization and can in many cases trigger unintended off-target effects. Crucially, the latter approaches rarely result in downregulation of gene expression exceeding 80–90%, which limits their utility for many applications. Although it is unlikely that any single approach can overcome all of the drawbacks of existing technologies, herein we sought to develop one that would optimize the ability to downregulate genes as completely as possible, preferably in a reversible manner.

Our group recently discovered that the lysosomal aspartyl protease, cathepsin D (CatD) [11,12,13] is, by several measures, the principal protease responsible for degrading intracellular pools of the amyloid β-protein (Aβ) [14], which accumulates abnormally in Alzheimer disease (AD) [15]. To follow up on this key finding, we have a long-term goal of developing methods for inducibly disrupting CatD, ultimately in AD mouse models. Conventional CatD-KO mice, unfortunately, are ill-suited for this purpose. On the one hand, deletion of both alleles of the gene for CatD (*CTSD*) produces an unrelated disease, neuronal ceroid lipofuscinosis (NCL), and also causes premature lethality by just 27 days of age [16]. On the other hand, deletion of just one *CTSD* allele yields no discernable effect on Aβ accumulation [14,17]. We therefore aimed to develop a system capable of completely deleting *CTSD*, if necessary, but ideally one also capable of achieving near-total disruption of CatD expression in a titratable and a reversible manner. Given that CRISPRi or RNAi rarely achieve near-complete downregulation of target genes, a novel approach was required.

Here we describe the design, development, and testing of a versatile, dual-function approach to disrupting gene expression, which we apply to the regulation of murine *CTSD*. Using CRISPR/Cas9-assisted homologous recombination, we inserted a tetracycline response element (TRE) together with LoxP sites into the 5′ region of the *CTSD* gene within mouse embryonic fibroblasts (MEFs). As we show, this “TRE-Lox” system permits genetic deletion of *CTSD* through traditional Cre recombinase-based approaches and—critically—also permits drug-inducible, titratable, and reversible downregulation by as much as 98%. The rapid kinetics and reversible nature of the TRE-Lox system are ideally suited for future cell-based studies on various aspects of CatD biology and, if implemented in mouse models, is expected to greatly improve our understanding of the role of CatD dysregulation in AD and other neurodegenerative disorders.

## 2. Results

**Figure 1:**
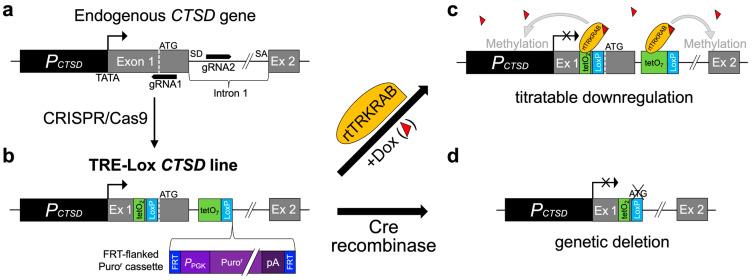
Overview of the design and dual-functionality of the TRE-Lox system. (**a**) Overall structure of the 5′ end of the murine cathepsin D (CatD) gene (*CTSD*) and its promoter region (*P_CTSD_*_,_ dark gray), indicating the relative position of the two gRNAs (black arrows) used for CRISPR/Cas9-assisted homologous recombination. Note the placement of the TATA box (TATA) very close to the main transcription start site (TSS) (right-angle arrow), the presence of the initiation codon (ATG, dashed white line) within Exon 1 (Ex 1, light gray), and the presence of a splice donor (SD) and splice acceptor (SA) flanking Intron 1 (black line). (**b**) Structure of the TRE-Lox knock-in (KI) insert, illustrating the relative positions of the two tet-operons (tetO_2_, green) and one LoxP site (LoxP, light blue) within the 5′ untranslated region (5′UTR) and, within Intron 1, a tetracycline response element (TRE) comprised of seven tetO repeats (tetO_7_) and the second LoxP site. The relative placement of the puromycin resistance cassette (Puro^r^, purple) flanked by two FRT sites (FRT, dark blue), which is excisable by Flp recombinase, is depicted using a curly bracket. (**c**) Downregulation of *CTSD* via the action of rtTRKRAB acting on the TRE-Lox insert. In the presence of Dox (red triangles), rtTRKRAB binds to the tetO repeats within both the 5′UTR and Intron 1, triggering methylation of histones in a radius of 2–3 kb, thereby remodeling the chromatin and silencing the *CTSD* gene. (**d**) Genetic deletion of *CTSD* via the action of Cre recombinase on the TRE-Lox insert. The figure depicts the end result of Cre-mediated recombination of the TRE-Lox KI insert, which causes removal of the initiation codon, the first portion of the coding region of Exon 1 encoding the signal peptide of CatD, and the 5′ end of Intron 1.

As a novel means of inducibly disrupting CatD expression either completely or partially, we designed a dual-purpose technology (Figure 1a–d) that combines the conventional Cre-Lox recombination approach [18] with a well-established system for triggering reversible histone methylation at a specific genomic locus in a drug-inducible manner [19,20]. The latter system, as adapted here, requires: (1) multiple tet-operon (tetO) DNA repeats at the targeted locus, collectively referred to as a tetracycline response element (TRE); (2) expression of a reverse-tetracycline transrepressor (rtTR) that binds to tetO repeats only in the presence of tetracycline (or, more typically, its more stable derivative, doxycycline (Dox)); and (3) a tag on the rtTR protein that recruits histone methylation machinery, such as the Krüppel-associated box (KRAB) domain [21]. In the presence of Dox, the rtTRKRAB fusion protein binds to tetO sequences, triggering localized histone methylation (specifically H3K9 trimethylation [20]) and consequent chromatin remodeling in a region 2–3 kb around the TRE [22] (Figure 1c). If positioned suitably within the target gene, this chromatin remodeling will result in partial to complete silencing of gene expression [23,24]. Notably, in many (but not all) systems using this approach, the histone methylation is reversible upon withdrawal of Dox, thereby permitting transient downregulation of target genes [23,25].

To develop a single system suitable for both approaches, we designed a knock-in (KI) construct for inserting both TRE and LoxP sequences into the endogenous murine *CTSD* gene. To maximize their potential effectiveness, we elected to insert these elements as close as possible to the transcription start sites (TSSs) of the *CTSD* gene. The endogenous murine *CTSD* promoter (Figure 1a; Supplementary Materials Figure S1) features a canonical TATA box that results in transcription beginning at multiple, closely spaced TSSs [26]. Exon 1, moreover, encodes both the complete 5′ untranslated region (5′UTR) and the initiation codon (Figure 1a; Supplementary Materials Figure S1). Taking these features into account, our final design incorporates the following elements: (1) two tetO repeats and a LoxP site within the 5′UTR of *CTSD*; (2) seven tetO repeats with optimized spacers (i.e., a 3rd-generation TRE [27]) within the first intron; and (3) a second LoxP site immediately downstream (Figure 1b; Supplementary Materials Figure S2). Collectively, we refer to this dual-function system as “TRE-Lox” technology.

To accelerate the evaluation of this system in cultured cells, we also included a puromycin resistance (Puro^r^) cassette, flanked by FRT sites, downstream of the other elements within our TRE-Lox KI insert (Figure 1b; Supplementary Materials Figure S2). Though ~1.7 kb in size, the Puro^r^ cassette could be readily excised by transient expression of Flp recombinase, leaving only a single 34-bp FRT site within Intron 1 (Figure 1b; Supplementary Materials Figure S3).

Placement of the two tetO repeats in the 5′UTR, by virtue of their proximity to the TATA box makes it theoretically possible that binding of rtTR alone (e.g., without the KRAB domain) might be sufficient to block transcription—something we explicitly test (see below)—merely by steric interference with transcriptional machinery. If this were the case, it would provide for the very rapid manipulation of *CTSD* expression and also overcome the potential for irreversible promoter methylation that KRAB can sometimes induce [28]; thus, it was considered worthy of testing. Notably, the elements within the 5′UTR add only 93 bp of additional sequence, reducing the possibility of disrupting endogenous expression. The complete predicted DNA sequences after insertion of the TRE-Lox KI insert into the *CTSD* gene (Supplementary Materials Figure S2), after deletion of the FRT-flanked Puro^r^ cassette by Flp recombinase (Supplementary Materials Figure S3), and after deletion of the LoxP-flanked regions of the *CTSD* gene by Cre recombinase (Supplementary Materials Figure S4) are provided in the Supplementary Materials.

To characterize this system in cells, we elected to use CRISPR/Cas9-assisted homologous recombination [29] to insert our TRE-Lox KI insert into the murine *CTSD* gene in C57Bl/6J MEFs. To ensure proper placement of the KI insert, and to also make it more likely that we might obtain some alleles that functionally delete *CTSD* via nonhomologous DNA end joining (NHEJ), we chose to use two gRNAs to effect homologous recombination [30]. Using the gRNA prediction tool CHOPCHOP [31], we found two well-positioned gRNAs in the target region, one that cuts just upstream of the initiation codon within Exon 1, and another that cuts 56 nt downstream of the Exon1/Intron 1 junction (Figure 1a; Supplementary Materials Figure S1). This placement allowed us to insert the two tetO sites and first LoxP site very close to the initiation codon (Figure 1b; Supplementary Materials Figure S2), thereby minimizing the possibility of disturbing the secondary structure of the 5′UTR.

In practice, to achieve homologous recombination, we used a multiplexed vector that expresses both gRNAs via separate polymerase III promoters and simultaneously expresses Cas9 via a mammalian promoter [32]. MEFs were co-transfected with this single vector together with linear dsDNA encoding the TRE-Lox KI insert, plus an extra ~50 nucleotides of flanking homology sequence on each end (see Materials and Methods and Supplementary Materials Figure S2), which were then selected in puromycin to obtain stable clones.

**Figure 2:**
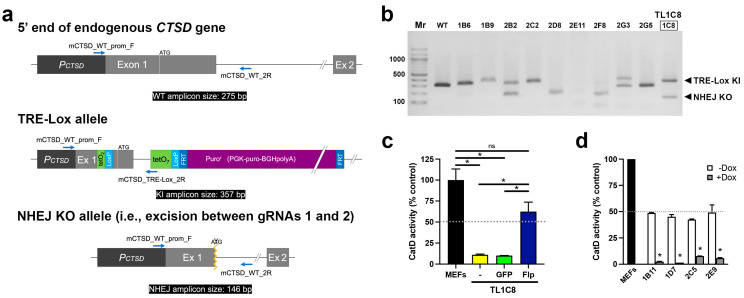
Development and characterization of TRE-Lox KI cell lines targeting the endogenous *CTSD* gene of mouse embryonic fibroblasts (MEFs). (**a**) Overview of the genotyping strategy, including the relative position of different primers (blue arrows) and the predicted sizes of different PCR amplicons (black boxes) used to distinguish different possible outcomes of insertion of the TRE-Lox construct via Cas9-assisted homologous recombination. The main possibilities include (but are not limited to) the following: (1) the unmodified endogenous murine *CTSD* allele (top); (2) insertion of the TRE-Lox KI insert as designed (middle); and (3) nonhomologous DNA end joining (NHEJ) resulting (in this case) in the excision of the segment of DNA between gRNA1 and gRNA2 (bottom, see Supplementary Materials Figure S1). (**b**) Genotyping of a subset of clones obtained after selection of individual puromycin-resistant clonal lines. The two bands within clone 1C8 (referred to as TL1C8) were excised, sequenced, and confirmed to be amplified from one allele carrying the TRE-Lox KI insert (upper band) and another allele featuring NHEJ, which results in functional knock-out (KO) of *CTSD* (sequences provided in Supplementary Materials Figure S5a–c). (**c**) CatD proteolytic activity in wild-type MEFs, or TL1C8 cells transiently transfected with empty vector (yellow), GFP (green) or Flp recombinase (dark blue). Note the low level of CatD activity in TL1C8 cells, which is reversed by transfection with Flp recombinase, resulting in activity close to the expected value of 50% of wild-type MEFs (gray dotted line). (**d**) CatD activity in several clonal lines of TL1C8-Flp cells stably expressing rtTRKRAB incubated for 4 d in the absence or presence of Dox (100 ng/mL). Note how, in the absence of Dox (white columns), all tested clones harbor CatD activity that is very close to 50% of the levels within MEFs (gray dotted line), as expected, whereas in the presence of Dox (gray columns), CatD activity is greatly decreased. Data in (**c**,**d**) are mean ± SEM of 4 replicates. * *p* < 0.05; ns = nonsignificant.

Once expanded, individual stable cell lines were genotyped by PCR (Figure 2). Multiple genotypes were obtained (Figure 2b), reflecting the fact that several possible outcomes could occur (e.g., wild-type, several types of NHEJ, or KI) in either or both *CTSD* alleles within each clone (Figure 2a). One clone, dubbed TL1C8, showed particular promise (Figure 2b), so the PCR amplicons were excised and sequenced. Sanger sequencing of the upper band (~350 bp) confirmed that one allele had successfully integrated the TRE-Lox KI insert exactly as designed (Supplementary Materials Figure S5a). The lower band (~150 bp) proved to be amplified from a knock-out (KO) allele created by NHEJ, resulting in precise excision of the DNA sequence between the two gRNAs cut sites, without an insertion or deletion (Supplementary Materials Figure S5b–d). This clone was used for all downstream studies.

Although the individual elements within the TRE-Lox KI insert were designed to minimize the possibility of interfering with endogenous *CTSD* expression once integrated, the relatively large (~1.7–kb) FRT-flanked Puro^r^ cassette (Figure 1b; Supplementary Materials Figure S2) could conceivably disrupt expression of the *CTSD* gene. Consistent with this prediction, the TL1C8 cell line, either untransfected or transiently transfected with GFP alone, showed markedly reduced CatD activity relative to wild-type MEFs (Figure 2c). However, after transient transfection with Flp recombinase [33], CatD activity was restored to ~60% of that present in wild-type MEFs (Figure 2c), approximately the level predicted for a cell line with just one functional copy of *CTSD*.

This transiently transfected pool, referred to as TL1C8-Flp, was selected by cell sorting using a green fluorescent protein (GFP) tag on the Flp recombinase in our expression vector [33]; see Materials and Methods). TL1C8-Flp cells therefore consist mostly—but not entirely—of cells with the Puro^r^ cassette deleted. Consistent with this, PCR genotyping of this pool (Supplementary Materials Figure S6a) before and after transient transfection with Flp recombinase yielded a substantial—but not complete—reduction in the “Before Flp” PCR amplicon, and the exclusive appearance of the “After Flp” amplicon in Flp recombinase-transfected (GFP-positive) cells (Supplementary Materials Figure S6b), as expected from Flp-mediated deletion of the FRT-flanked Puro^r^ cassette in most cells.

The TL1C8-Flp pool was subsequently used for the generation of stable clones also expressing an optimized rtTRKRAB fusion protein. This optimized construct incorporates a single amino-acid substitution, V9I, that has been shown to increase the Dox sensitivity and maximal activation mediated by corresponding reverse-tetracycline transactivator (rtTA)-based systems [34]. We elected to test versions containing this mutation because our long-term ambition is to generate a mouse line permitting CatD downregulation within the brain, where it can be difficult to achieve high levels of Dox.

The vector expressing rtTRKRAB_V9I_ also co-expresses both G418 resistance and the red fluorescent protein, mCherry (see Materials and Methods). To generate stable clones, we transfected the stable pool of TL1C8-Flp cells with rtTRKRAB_V9I_, grew them in the presence of G418 (0.6 mg/mL) for 3 d, and then plated individual mCherry-positive cells in 96-well plates by cell sorting. Once expanded, these stable clones were split into two groups, one grown in the absence and one in the presence of Dox (100 ng/mL). After 4 d, these cells were harvested and evaluated for CatD activity. As illustrated in Figure 2d, we obtained multiple stable lines that, in the absence of Dox, all showed the predicted activity level (~50% of wild-type MEFs). Notably, incubation with Dox (100 ng/mL) for just 4 d resulted in average reductions in CatD activity of 90.3 ± 3.4% (*n* = 4) relative to the activity in each stable clone in the absence of Dox (Figure 2d).

**Figure 3:**
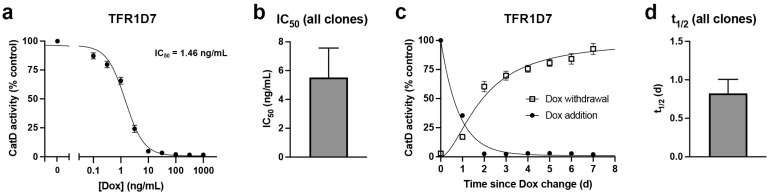
Detailed functional characterization of Dox-mediated downregulation of CatD activity made possible by the TRE-Lox system. (**a**) CatD activity as a function of Dox concentration in the TFR1D7 stable cell line. Cells were treated for 4 d with the indicated concentrations of Dox. The calculated IC_50_ is indicated. Note that for all Dox concentrations above 100 ng/mL, CatD activity is essentially completely abrogated. (**b**) IC_50_ values for three separate stable clones (see Supplementary Materials Figure S7). (**c**) Time course of CatD activity following addition or withdrawal of Dox (100 ng/mL) in TFR1D7 cells incubated for 7 d prior in the absence or presence of Dox (100 ng/mL), respectively. Data are mean ± SEM; *n* = 3. (**d**) Average half-life (t_1/2_) of the reduction in CatD activity after addition of Dox (100 ng/mL) to three separate stable clones (see Supplementary Materials Figure S8). Data are mean ± SEM; *n* = 3. Effects on CatD protein levels are shown in Supplementary Materials Figure S10.

To quantify more accurately the sensitivity of these clones to Dox, and also to determine the maximum achievable downregulation, we conducted Dox dose-response experiments. Clone TFR1D7, which showed the largest decrease (96.7%) in CatD activity following 4-d treatment with 100 ng/mL Dox (Figure 2d), exhibited a remarkably low IC_50_ of 1.46 ng/mL Dox (Figure 3a). Notably, in this paradigm, which also utilized 4-d treatments with Dox, the average reduction in CatD activity obtained for the three highest doses tested (100, 316, and 1000 ng/mL) was a remarkable 98.2 ± 0.14% (*n* = 9) relative to Dox-naïve TFR1D7 controls (see Figure 3a). Impressively, even a very low dose of just 10 ng/mL Dox produced a 95.2 ± 0.63% (*n* = 3) reduction in CatD activity (see Figure 3a). When combined with dose-response curves obtained for clones TFR1B11 (Supplementary Materials Figure S7a) and TFR2E9 (Supplementary Materials Figure S7b), the average IC_50_ for Dox-mediated downregulation was 5.65 ± 2.1 ng/mL (*n* = 3; Figure 3b).

To assess the kinetics of downregulation after addition and withdrawal of Dox, we performed time-course experiments using clone TFR1D7. Following administration of Dox (100 ng/mL) to Dox-naïve cells, downregulation of CatD occurred remarkably quickly, resulting in a calculated half-life (t_1/2_) averaging just 0.57 ± 0.05 d (Figure 3c). Time courses conducted on two other stable clones, TFR1B11 (Supplementary Materials Figure S8a) and TFR2E9 (Supplementary Materials Figure S8b) exhibited somewhat longer t_1/2_ values of 0.714 and 1.18 d, respectively, yielding an overall average of 0.82 ± 0.18 d (*n* = 3) for the three different clones tested (Figure 3d). The kinetics of restoration of CatD expression after Dox withdrawal, evaluated in TFR1D7 cells treated with 100 ng/mL Dox for the 7 d prior, proceeded more slowly (Figure 3c). Almost 2 d were required to achieve 50% restoration of CatD activity and 4 d to achieve 75% restoration (Figure 3c). Nevertheless, by 7 d after Dox withdrawal, CatD activity became statistically indistinguishable from Dox-naïve controls (92.7 ± 4.8% of controls; *n* = 3), showing that this system is indeed reversible.

Given the success of our system using rtTRKRAB, we wondered whether the binding of rtTR alone, in the absence of KRAB, might be sufficient to substantially downregulate *CTSD*. To test this, we generated a KRAB-free version of our rtTRKRAB/mCherry/G418^r^ expression construct, dubbed rtTR. As before, the TL1C8-Flp pool was transfected with rtTR or, as a control, mCherry alone. Pools of rtTR-transfected cells (along with mCherry-only transfected controls) as well as individual cells were isolated by cell sorting, selected in G418, and subjected to analysis after incubation with different concentrations of Dox for varying lengths of time. Despite testing both the stable pool and multiple stable clones in the presence of as much as 1000 ng/mL Dox for as long as 14 d, we obtained only modest reductions in CatD activity in pools (6.82 ± 2.1% in +Dox vs. -Dox; *n* = 4; Supplementary Materials Figure S9a) and in multiple stable clones (averaging 10.4 ± 2.2% *n* = 4; Supplementary Materials Figure S9b). These findings strongly suggest that, despite the close proximity of the two tetO sequences in the 5′UTR to the TATA box, mere binding of rtTR to these sequences is insufficient to achieve substantial downregulation of *CTSD* in this system.

**Figure 4:**
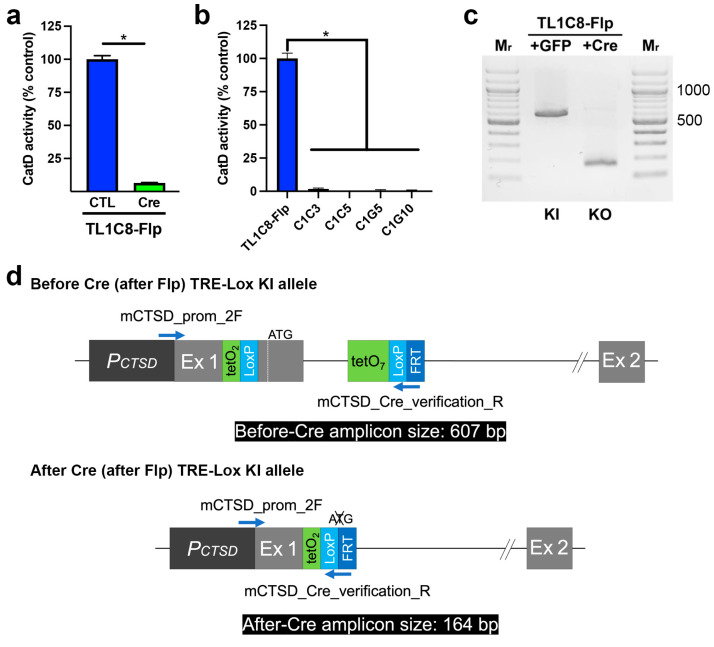
Functional and genotypic characterization of Cre-mediated genetic deletion of CatD made possible by the TRE-Lox system. (**a**) CatD activity in TL1C8-Flp cells transiently transfected with either GFP only (CTL, dark blue) or GFP-tagged Cre recombinase (Cre, green). Note that these are pools of GFP-positive cells selected by cell sorting 2 d after transfection. Data are mean ± SEM, *n* = 4, * *p* < 0.01. (**b**) CatD activity in multiple stable clones treated previously with Cre recombinase. Data are mean ± SEM, *n* = 4, * *p* < 0.01. (**c**) PCR genotyping of TL1C8-Flp cells before and after transfection with Cre recombinase. (**d**) Overview of the genotyping strategy based on the predicted consequences of Cre-mediated recombination of the TRE-Lox construct, including the relative position of different primers (blue arrows) and the predicted sizes of different PCR amplicons (black boxes) used to distinguish different possible outcomes.

Next, we assessed the functionality of the “Lox” portion of our dual-function TRE-Lox system (see Figure 1d). Consistent with expectations, transient transfection of the TL1C8-Flp pool with Cre-recombinase fused to GFP [33], followed by sorting 2 d later for GFP-positive cells, resulted in a 93.5 ± 0.37% reduction in CatD activity relative to control (GFP-transfected, GFP-positive sorted) cells (Figure 4a). Multiple stable clones derived from single cells showed essentially complete ablation of CatD activity, with KO cells showing an average reduction of 99.3 ± 0.41% (*n* = 4) (Figure 4b). As expected, PCR genotyping (Figure 4c) confirmed that Cre recombinase successfully deleted the DNA region between the LoxP sites, yielding PCR amplicons of the predicted sizes (Figure 4d).

Having established CatD-KO cell lines, we used these cells along with MEFs to help quantify CatD protein levels in TFR1D7 cells, both Dox-naïve and Dox-treated (Supplementary Materials Figure S10). Consistent with the CatD activity measurements (Figure 2b,c), CatD protein levels in TFR1D7 cells were ~50% of that in MEFs, as established using a standard curve of MEF protein (Supplementary Materials Figure S10a,b). As expected, the *CTSD* KO cell line C1C5 showed no detectable CatD protein (Supplementary Materials Figure S10a,b). We also conducted western blots on samples (5 µg/lane) from the time-course shown in Figure 3c. Consistent with these activity data, TFR1D7 cells grown for 7 d in the absence or presence of Dox (100 ng/mL) showed ample CatD protein and no detectable CatD protein, respectively (Supplementary Materials Figure S10c,d). As expected, Dox addition or withdrawal, respectively, to the latter cells for 6 d reversed the pattern of CatD expression essentially completely (Supplementary Materials Figure S10c,d).

## 3. Discussion

Here we describe the design, development, and quantitative characterization of a novel “TRE-Lox” system for versatile regulation of the murine *CTSD* gene. This system not only enables complete (irreversible) genetic deletion of CatD, like conventional Cre-Lox technology, but uniquely, it also permits titratable and reversible downregulation of the gene in a Dox-inducible manner.

A key advantage of this TRE-Lox approach is its ability to effect near-complete downregulation (~98%) in a Dox-dependent manner. This remarkable degree of effectiveness appears to depend on two unique features of the system. First, the design incorporates a total of nine tetO repeats: two in the 5′UTR portion of Exon 1, and seven in Intron 1. The effectiveness of these elements is likely attributable to their proximity to the TATA box and principal TSSs and, perhaps, also to the sheer number of tetO repeats. As implemented here, the incorporation of two tetO repeats (plus one LoxP site) within the 5′UTR of *CTSD* did not disrupt expression of the endogenous gene. Critically, whereas the seven tetO repeats within Intron 1 were separated by 17-bp spacers, the two within the 5′UTR were separated by only 2 bp, more closely resembling the configuration of the original *E. coli* Tn10 element upon which Tet-On/Off systems are based [3] (Supplementary Materials Figure S2). The small size of this two-tetO element may therefore account for the lack of disruption to endogenous *CTSD* expression that we observed. We emphasize, however, that addition of this element into the 5′UTR of other target genes might nevertheless disrupt their expression; hence, empirical testing is essential.

The second novel feature of this Dox-regulatable system was the use of rtTRKRAB incorporating the V9I mutation, which is located within the DNA-binding domain of the rtTR portion. When included with other performance-enhancing mutations in the corresponding transactivator (rtTA) system (a.k.a., Tet-On) [27,34,35], the V9I mutation results in 7-fold more activation at high Dox levels and 100-fold more Dox sensitivity than earlier versions of rtTA (Tet-On Advanced, Takara Bio USA, San Jose, CA, USA) [34]. Despite also outperforming the best system currently available (e.g., Tet-On 3G, Takara Bio USA, San Jose, CA, USA), the V9I mutation has been excluded from commercial vectors, presumably because low-level leakiness is observed when Tet-On components are present episomally following transient transfection [34]. Notably, however, such leakiness does not occur when rtTA_V9I_ and its targets are stably integrated into the genome [34]. Consequently, we elected to incorporate this mutation given our long-term goal of generating transgenic mouse lines suitable for manipulating *CTSD* in brain, where obtaining high levels of Dox can be challenging. Confirming the findings for this mutation incorporated into rtTA, we observed no leakiness and very potent sensitivity to Dox (low-ng/mL IC_50_s) with rtTRKRAB_V9I_. To our knowledge, this is the first time the V9I mutation has been tested in rtTR-based systems.

The peculiar placement of the two LoxP sites in our system—one within an exon and one within an intron—is an atypical use of the Cre-Lox system, which typically involves flanking one exon with two LoxP sites, resulting in a so-called “floxed” exon. In our case, Cre-based recombination resulted in the removal of the initiation codon, together with portions of DNA encoding the signal peptide of CatD. The deletion of one splice donor, but not a corresponding splice acceptor was unusual but unavoidable given our desire to place the LoxP elements as closely as possible to the TSSs. Despite its unorthodox nature, this design proved entirely effective for achieving genetic deletion and, as such, might inspire alternative uses for the placement of LoxP sites for conditional regulation of target genes.

Our decision to develop the TRE-Lox system was motivated by difficulties we encountered with an alternative approach we explored to achieve neuron-specific downregulation of CatD (ultimately, in mice). Briefly, we developed and tested a transgene comprised of a neuron-specific promoter driving Dox-regulatable expression of micro RNA-based, short hairpin RNAs targeting *CTSD*, which were encoded within and spliced from the introns of the transgene [36,37]. Despite extensive optimization, we were only able to achieve a maximum of ~80% knockdown of CatD using this system and only then in the presence of relatively high concentrations of Dox (1000 ng/mL). We also considered various CRISPRi-based approaches, but we could identify exceedingly few examples of near-complete downregulation of a target gene using these approaches, even those deploying multiple gRNAs [38,39].

Although we succeeded in achieving our desired goals with the TRE-Lox system in cells, a distinct disadvantage of this approach when implemented in vivo is the need to also express rtTRKRAB through a separate system (e.g., via a virus or a separate transgenic mouse line). Compared to single-transgene systems based on CRISPRi or shRNAs, this greatly increases the breeding required to develop mouse lines both homozygous for the TRE-Lox KI modification and also expressing rtTRKRAB, a problem that is exacerbated further when modeling diseases that require other transgenes to be expressed simultaneously [40]. In addition, the TRE-Lox system is comparable to conventional Cre-Lox technology in terms of the time and cost involved, although these concerns are mitigated by widespread access to CRISPR-based technologies.

In sum, the TRE-Lox system has proved to be a versatile way to disrupt *CTSD* expression, either through irreversible genetic deletion or through Dox-regulatable downregulation, in the latter case in a titratable, and fully reversible manner. This dual-purpose approach is expected to expand our understanding of the biology of CatD in particular and, if applied successfully to other target genes, may prove to be a generalizable way to disrupt gene expression in a more versatile manner than is currently possible via conventional, single-purpose approaches.

## 4. Materials and Methods

### 4.1. Reagents

Except where otherwise noted, all reagents were purchased from ThermoFisher Scientific (Waltham, MA, USA).

### 4.2. Targeted Modification of the CTSD Gene 

To insert the TRE-Lox system into the endogenous murine *CTSD* gene, we performed Cas9-assisted homologous recombination with two guide RNAs (gRNAs), selected using CHOPCHOP [31] on the GRCm38/mm10 draft of the *mus musculus* genome, and a linear dsDNA targeting insert as described [30]. Briefly, we first assembled a template vector for the knock-in (KI) insert by using NEBuilder HiFi DNA Assembly (New England Biolabs, Waltham, MA, USA) to combine overlapping PCR amplicons derived from appropriate sources. Relevant regions (and their sources) were as follows: the 3′ end of Exon 1 and the 5′ portion of Intron 1 of murine *CTSD* (from C57Bl6/J mouse tail DNA); tetO_2_ (from Addgene plasmid #113892 [41]); TRE 3G and, separately, an FRT-flanked puromycin resistance cassette (both from Addgene plasmid #156430 [42]). LoxP sequences were included within the primers used for PCR amplification. Once assembled, the linear dsDNA targeting KI insert was obtained by PCR amplification with primers containing ~50 bp of flanking homology sequence and also featuring phosphorothioate bonds at the two 5′-most linkages to improve the stability of the amplicon after transfection (see Supplementary Materials Table S1; Supplementary Materials Figure S2). A single vector expressing the two gRNAs and Cas9 was assembled from pX330S-2 (Addgene plasmid #58778) and px330A-1x2 (Addgene plasmid #58766) according to established protocols [32]. All primers and oligonucleotide sequences are provided in Supplementary Materials Table S1.

Wild-type C57Bl/6J SV40 large T antigen-immortalized mouse embryonic fibroblasts (MEFs; Cat #CRL-2907; ATCC, Manassas, VA, USA) grown to near confluency in 6-well plates were transfected with the 2-gRNA/Cas9 vector (2.0 µg) and the linear, PCR-amplified targeting KI insert (0.4 µg) using Lipofectamine™ 3000 according to manufacturer’s recommendations (ThermoFisher). After 24 h the cells were passaged into T75 flasks and selected in puromycin (4 µg/mL) then, 7–10 d later, single puromycin-resistant cells were plated into 96-well plates by fluorescence-activated cell sorting (FACS) using a BD FACSAria™ II Cell Sorter; BD BioSciences, Franklin Lakes, NJ, USA). Individual clones were expanded and genotyped as described in Figure 1a.

To remove the FRT-flanked puromycin resistance cassette, TL1C8 cells were subsequently transfected with an enhanced form of Flp recombinase (Flpe) fused with GFP (pCAG-Flpe:GFP; Addgene plasmid #13788 [33]), and pools of Flpe (and GFP-only control)-transfected cells were isolated by FACS. To achieve Cre-mediated deletion, TL1C8-Flp cells were transfected with pCAG-Cre:GFP (Addgene plasmid #13776 [33]), and GFP-positive cells were selected by FACS.

### 4.3. Cloning of the rtTRKRAB_V9I_ and rtTR Expression Constructs and Generation of Stable Cell Lines Derived from Them

NEBuilder HiFi DNA Assembly (New England Biolabs, Ipswich, MA) was used to clone the rtTR:KRAB fusion protein containing the activity-enhancing V9→I mutation into the multiple cloning site of pICherryNeo (Addgene plasmid #52119), which uses the CMV promoter to drive expression of a gene of interest together with mCherry (via an IRES) while also expressing neomycin/G418 resistance (G418^r^). Briefly, the 3rd-generation reverse-tetracycline transactivator (Tet-ON^®^ 3G) also containing the V9I mutation (omitting the VP16 activator domain) was amplified from pAC-V16-EGFP (Addgene plasmid #122033 [43]) using appropriate primers (Supplementary Materials Table S1). The KRAB domain was amplified similarly from pLVPRT-tTR-KRAB (Addgene Plasmid #11648 [37]) to permit an in-frame fusion with rtTR. The preceding amplicons were assembled into pICherryNeo digested with NheI and EcoRI using NEBuilder (New England Biolabs, Ipswich, MA). To generate the same vector with the KRAB domain deleted (rtTR), inverse PCR was conducted with appropriate primers (Supplementary Materials Table S1). All vectors were confirmed to be correct by DNA sequencing.

Stable cell lines expressing rtTRKRAB or rtTR were generated by transfecting a pool of Flpe-transfected TL1C8 cells with the corresponding vectors then, 24 h later, plating single cells expressing mCherry in 96-well plates by cell sorting. Resulting clones were selected for stable expression by growth in G418 (0.6 mg/mL).

### 4.4. Cell Culture 

MEFs were maintained at 37 °C in a humidified incubator supplemented with 5% CO_2_ in DMEM containing GlutaMAX^®^ supplemented with 10% Tet System Approved Fetal Bovine Serum (hereafter FBS; Takara Bio USA, San Jose, CA, USA), 100U/mL penicillin, and 100 µg/mL streptomycin.

### 4.5. CatD Activity Assays

CatD proteolytic activity was assessed by continuous monitoring of the digestion of an internally quenched, fluorogenic peptide substrate (Mca-GKPILFFRLK(Dnp)-r-NH_2_) according to manufacturer’s recommendations (InnoPep, Inc. San Diego, CA, USA). In a typical reaction, near-confluent monolayers of MEFs in 6-well plates were detached by brief digestion with trypsin-EDTA, washed once in DMEM/10% FBS, then two times in PBS. Pelleted cells were lysed in 300 µL Lysis Buffer (40 mM NaOAc, 0.1% CHAPS, pH 3.5), incubated on ice for 10 min, then centrifuged at 21,000× *g* for 1.5 min. The resulting supernatant was transferred to a new microcentrifuge tube, mixed, then loaded in quadruplicate onto non-binding surface, round-bottom, low-volume, black 384-well microplates (Corning^®^ CLS4514; 10 µL/well). The concentration of the supernatant was estimated by A280 quantification using a NanoDrop^®^ ND-1000 UV-Vis Spectrophotometer. For each sample, a subset of wells was supplemented with the CatD inhibitor pepstatin A (PepA; 1 µM final conc.; MilliporeSigma, Inc., Burlington, MA, USA). To initiate the reactions, 10 µL/well of Reaction Buffer (100 mM NaOAc, 0.2 M NaCl, pH 3.5) supplemented with fluorogenic substrate (4 µM final conc.) was added to each well. Plates were immediately loaded into a microplate reader (Gemini EM, Molecular Devices, LLC, San Jose, CA, USA), and fluorescence (λ_ex_ = 328 nm, λ_em_ = 393 nm) was read continuously every 15 s for ≥10 min. Proteolytic activity was calculated as follows: the slopes of the initial, linear portions of progress curves from each well, obtained using SoftMax Pro (v. 5.0; Molecular Devices, LLC, San Jose, CA, USA), were exported into Microsoft Excel; the slopes of PepA-containing wells from each sample were subtracted; then activity was normalized to its A280 reading. Data were expressed as a percentage of activity relative to that within MEFs or the parental cell line (e.g., TL1C8).

### 4.6. Western Blotting

For protein collection, MEFs were grown to near-confluency in T75 flasks, harvested by trypsin/EDTA treatment, washed once in DMEM/10% FBS, then washed twice in PBS. The resulting cell pellets were resuspended in 6 vol Protein Lysis Buffer (50 mM Tris-HCl, 1% Triton X-100, pH 7.4) supplemented with 1x Halt™ Protease Inhibitor Cocktail, incubated on ice for 30 min, then centrifuged at 5000× *g* at 4 °C for 5 min. The resulting supernatant was aliquoted into new tubes and stored at −80 °C. Prior to western blotting, the protein content was quantified using the BCA Protein Assay according to manufacturer’s recommendations (ThermoFisher, Waltham, MA, USA,). In some cases, we analyzed protein obtained for activity assays as described above. Depending on the source, 5–20 µg of protein was combined with NuPAGE LDS Loading Buffer plus reducing agent, heated to 70 °C for 10 min to denature proteins, loaded on NuPAGE 4–12% Bis-Tris Precast Protein Gels, electrophoresed in MES running buffer then transferred to 0.2-μm nitrocellulose membranes using a Trans-Blot Turbo Transfer System (Bio-Rad Laboratories, Hercules, CA, USA). Protein-bound membranes were washed in Tris-buffered saline supplemented with 0.1% Tween-20 (TBS-T), blocked with 5% milk in TBS-T for 1 h at room temperature, then incubated O/N at 4 °C with primary rabbit anti-CatD antibody (Cat. No. PIPA579094; 1:2000) or HRP-conjugated anti-GAPDH antibody (Cat. No. MA515738HRP; 1:100,000). After extensive washing with TBS-T, the blot probed with anti-CatD antibody was incubated with HRP-conjugated secondary antibody (Cat. No. ab97051; Abcam; 1:20,000) for 1 h, washed again with TBS-T, then the blot was visualized by enhanced chemoluminescence and exposure to X-ray film as described [14].

## Data Availability

Data are contained within the article and Supplementary Material.

## Supplementary Materials

The following supporting information can be downloaded at: https://www.mdpi.com/article/10.3390/ijms24076745/s1.
