# Supplementary material for: A Dual-Function “TRE-Lox” System for Genetic Deletion or Reversible, Titratable, and Near-Complete Downregulation of Cathepsin D
Source: Int J Mol Sci. 2023 Apr 4;24(7):6745. doi: 10.3390/ijms24076745 (PMC10095275; doi:10.3390/ijms24076745)
Supplement: Supplementary file 1 [file ijms-24-06745-s001.zip › ijms-2294935-supplementary.pdf]

Supplemental Materials
for

A Dual-Function "TRE-Lox" System for Genetic Deletion or Reversible, Titratable, and Near-Complete Downregulation of Cathepsin D

Heather M. Terron[1], Derek S. Maranan[1,2], Luke A. Burgard[1,2], Frank M. LaFerla[1,2], Shelley Lane[1] and Malcolm A. Leissring[1,*]

[1] Institute for Memory Impairments and Neurological Disorders, University of California, Irvine, Irvine, CA 92697, USA
[2] Department of Neurobiology and Behavior, University of California, Irvine, Irvine, CA 92697, USA

[*] Correspondence: m.leissring@uci.edu

Contents

pp.	Fig./Table	Title
2	Fig. S1	Targeted region of murine *CTSD* gene highlighting key elements.
3-4	Fig. S2	Targeted region after insertion of TRE-Lox KI insert with Puro^r cassette.
5	Fig. S3	Targeted region after Flp recombination.
6	Fig. S4	Targeted region after genetic deletion (recombination w/ Flp and Cre).
7	Fig. S5	Genotyping of TL1C8 line by sequencing.
8	Fig. S6	PCR verification before vs. after Flp-mediated recombination.
9	Fig. S7	Dox dose-response curves for stable clones TFR1B11 and TFR2E9.
10	Fig. S8	Time courses of CatD downregulation in clones TFR1B11 and TFR2E9 after addition of Dox (100 ng/mL).
11	Fig. S9	Effect of rtTR on CatD activity in the presence and absence of Dox (1000 ng/mL) in transient pools and stable clones of TL1C8-Flp cells.
12	Fig. S10	Steady-state CatD protein levels in MEFs vs. TFR1D7 cells and in response to Dox addition and withdrawal, using CatD-KO line C1C5 as a control.
13	Table S1	Primers used in this study.

Supplementary Figure S1. Targeted region of murine *CTSD* gene highlighting key elements.

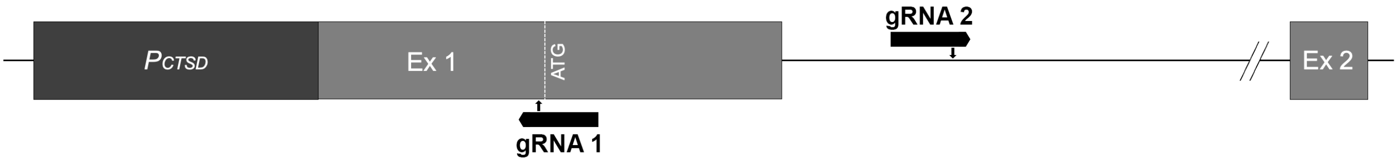

Promoter:	lower-case gray
TATA Box:	underlined
Transcript. start:	↱
EXON 1:	UPPER-CASE BLACK
Introns:	lower-case black
ATG site:	OUTLINED
gRNA seqs:	**BOLD BLACK**
gRNA orientation:	horizontal arrows
PAM seqs:	Yellow highlight
Cut site:	⇑

...acccgttgagggccaaacaagcgggtcagctgactccgcgggactgc

↱

ggcgtcatcctgcctATAAGCCGGCGACCTCTGGCTTTAAGCTTTGCTCT

CTTCGGG**CCGCCGCGACCATGAAGACTCCC**GGCGTCTTGCTGCTCATTCT

←-----⇑------------------‹

CGGCCTCCTGGCTTCGTCCTCCTTCGCGATTATCAGgtgaggacccgctc

›------------------⇓-----→

tgggtccggagatgcggggctcgtc**acctggagtgccgcgtgctccgg**cc

gtgctggaatgcacctgtgcacccagcgcagccttcctcaggtccc...

Supplementary Figure S2. Targeted region after insertion of TRE-Lox KI insert with Puro^r cassette.

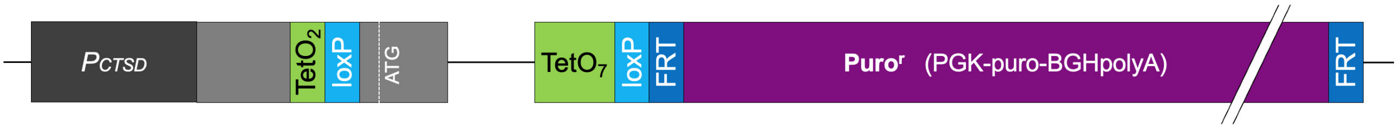

Flanking seq: gray text
Homology Arms: *purple italic*
TATA Box: underlined
EXONS: UPPER CASE
Introns: lower case black
KI region: **bold**
ATG sites: OUTLINED
Stop codons: black box
Spacer seqs: gray highlight
TetO repeats: green highlight
LoxP sites: light blue highlight
FRT sites: blue highlight
PGK-Puro^r-pA: violet highlight

...gttcgggtcgccccgcccctcgcccgcgtctcacgtgacccgttgag
ggccaaacaagcgggtcagctgactccgcgggactgcggcgtcatcctgc
ctATAAGCCGGCGACCTCTGGCTTTAAGCTTTGCTCTCTTCGGCCGCCG
CAAGCAGAGCTCTCCCTATCAGTGATAGAGATCTCCCTATCAGTGATAGA
GATCGTCGATAACTTCGTATAAAGTATCCTATACGAAGTTATAGCCGCGA
CCATGAAGACTCCCGGCGTCTTGCTGCTCATTCTCGGCCTCCTGGCTTCG
TCCTCCTTCGCGATTATCAGgtgaggacccgctctgggtccggagatgcg
gggctcgtcacctggagtgccgcg**tcttcactcgagtttactccctatca**
gtgatagagaacgtatgaagagtttactccctatcagtgatagagaacgt
atgcagactttactccctatcagtgatagagaacgtataaggagtttact
ccctatcagtgatagagaacgtatgaccagtttactccctatcagtgata
gagaacgtatctacagtttactccctatcagtgatagagaacgtatatcc
agtttactccctatcagtgatagagaacgtatgtcgaggtagataacttc
gtataaagtatcctatacgaagttatgtagtaataactagtcgaagttcc
tattctctagaaagtataggaacttcaggtctgaagaggagtttacgtcc
agccaagctagcttggctgcaggtcgtcgaaattctaccgggtaggggag
gcgctttcccaaggcagtctggagcatgcgctttagcagccccgctggg
cacttggcgctacacaagtggcctctggcctcgcacacattccacatcca
ccggtaggcgccaaccggctccgttctttggtggccccttcgcgccacct

Supplementary Figure S2. Targeted region after insertion of TRE-Lox KI insert with Puro^r cassette (cont'd).

tctactcctcccctagtcaggaagttcccccccgccccgcagctcgcgtc
gtgcaggacgtgacaaatggaagtagcacgtctcactagtctcgtgcaga
tggacagcaccgctgagcaatggaagcgggtaggcctttggggcagcggc
caatagcagctttgctccttcgctttctgggctcagaggctgggaagggg
tgggtccgggggcgggggcgggcgcccgaaggtcctccggaggcccggcat
tctgcacgcttcaaaagcgcacgtctgccgcgctgttctcctcttcctca
tctccgggcctttcgacctgcagcctgttgacaattaatcatcggcatag
tatatcggcatagtataatacgacaaggtgaggaactaaaccatgaccga
gtacaagcccacggtgcgcctcgccacccgcgacgacgtccccagggccg
tacgcaccctcgccgccgcgttcgccgactaccccgccacgcgccacacc
gtcgatccggaccgccacatcgagcgggtcaccgagctgcaagaactctt
cctcacgcgcgtcgggctcgacatcggcaaggtgtgggtcgcggacgacg
gcgccgcggtggcggtctggaccacgccggagagcgtcgaagcgggggcg
gtgttcgccgagatcggcccgcgcatggccgagttgagcggttcccggct
ggccgcgcagcaacagatggaaggcctcctggcgccgcaccggcccaagg
agcccgcgtggttcctggccaccgtcggcgtctcgcccgaccaccagggc
aagggtctgggcagcgccgtcgtgctccccggagtggaggcggccgagcg
cgccggggtgcccgccttcctggagacctccgcgccccgcaacctcccct
tctacgagcggctcggcttcaccgtcaccgccgacgtcgaggtgcccgaa
ggaccgcgcacctggtgcatgacccgcaagcccggtgcctgaggggatca
attctctagagctcgctgatcagcctcgactgtgccttctagttgccagc
catctgttgtttgcccctcccccgtgccttccttgaccctggaaggtgcc
actcccactgtcctttcctaataaaatgaggaaattgcatcgcattgtct
gagtaggtgtcattctattctggggggtggggtggggcaggacagcaagg
gggaggattgggaagacaatagcaggcatgctggggatgcggtgggctct
atggcttctgaggcggaaagaaccagctgggctcgactagagcttgcgg
aacccttcgaagttcctattctctagaaagtataggaacttcggcgcgcc
atttcggccgtgctggaatgcacctgtgcacccagcgcagccttcctcag
ggtcccacagactatgggcgagacactcaaaaggcaggggtctcctggg
cccctctccactcctatgactcctatggtcgaacaggagggtacagt...

Supplementary Figure S3. Targeted region after Flp recombination to remove Puro^r cassette.

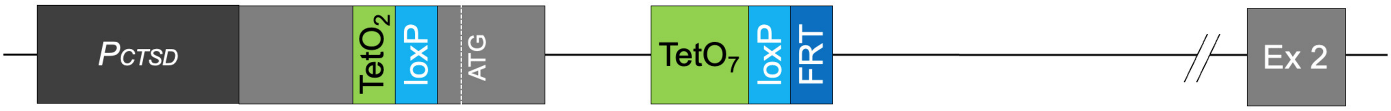

Flanking seq: gray text
Homology Arms: *purple italic*
TATA Box: underlined
EXONS: UPPER CASE
Introns: lower case black
KI region: **bold**
ATG sites: OUTLINED
Stop codons: black box
Spacer seqs: gray highlight
TetO repeats: green highlight
LoxP sites: light blue highlight
FRT sites: blue highlight

...gttcgggtcgccccgcccctcgcccgcgtctcacgtgacccgttgag
ggccaaacaagcgggtcagctgactccgcgggactgcggcgtcatcctgc
ctATAAGCCGGCGACCTCTGGCTTTAAGCTTTGCTCTTCGGGCCGCCG
CAAGCAGAGCTCTCCCTATCAGTGATAGAGATCTCCCTATCAGTGATAGA
GATCGTCGATAACTTCGTATAAAGTATCCTATACGAAGTTATAGCCGCGA
CCATGAAGACTCCCGGCGTCTTGCTGCTCATTCTCGGCCTCCTGGCTTCG
TCCTCCTTCGCGATTATCAGgtgaggacccgctctgggtccggagatgcg
gggctcgtcacctggagtgccgcgtcttcactcgagtttactccctatca
gtgatagagaacgtatgaagagtttactccctatcagtgatagagaacgt
atgcagactttactccctatcagtgatagagaacgtataaggagtttact
ccctatcagtgatagagaacgtatgaccagtttactccctatcagtgata
gagaacgtatctacagtttactccctatcagtgatagagaacgtatatcc
agtttactccctatcagtgatagagaacgtatgtcgaggtagataacttc
gtataaagtatcctatacgaagttatgtagtaataactagtcgaagttcc
tattctctagaaagtataggaacttcgaagttcctattctctagaaagta
taggaacttcggcgcgccattt*cggccgtgctggaatgcacctgtgcacc*
*cagcgcagccttcctcagggtc*ccacagactatgggcgagacactcaaa
aggcaggggtctcctgggcccctctccactcctatgactcctatggtcga
acaggagggtacagt...

Supplementary Figure S4. Targeted region after genetic deletion (recombination w/ Flp and Cre).

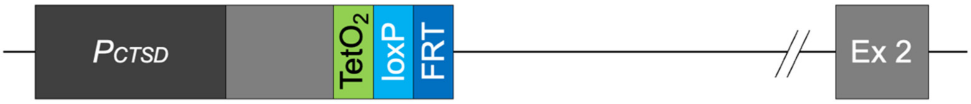

Flanking seq: gray
Homology Arms: *purple italic*
TATA Box: underlined
KI region: **bold black**
ATG sites OUTLINED UPPER CASE
Stop codons: black box
Spacer seqs: gray highlight
TetO repeats: green highlight
LoxP site: light blue highlight
FRT site: blue highlight

...gttcgggtcgccccgcccctcgcccgcgtctcacgtgacccgttgag
ggccaaacaagcgggtcagctgactccgcgggactgcggcgtcatcctgc
ct*ataagccggcgacctctggctttaagctttgctctcttcggccgccg*
*cg***gtggaattgctctccctatcagtgatagagatctccctatcagtgata**
gagatcgtcgataacttcgtataaagtatcctatacgaagttatgtagta
ataactagtcgaagttcctattctctagaaagtataggaacttcggcgcg
ccattt*ctccggccgtgctggaatgcacctgtgcacccagcgcagccttc*
*ctcagg*gtcccacagactatgggcgagacactcaaaggcaggggtctc
ctgggcccctctccactcctatgactcctatggtcgaacaggagggt...

Supplementary Figure S5. Genotyping of TL1C8 line by sequencing.

a. Alignment of sequencing of upper, TRE-Lox KI PCR amplicon from TL1C8

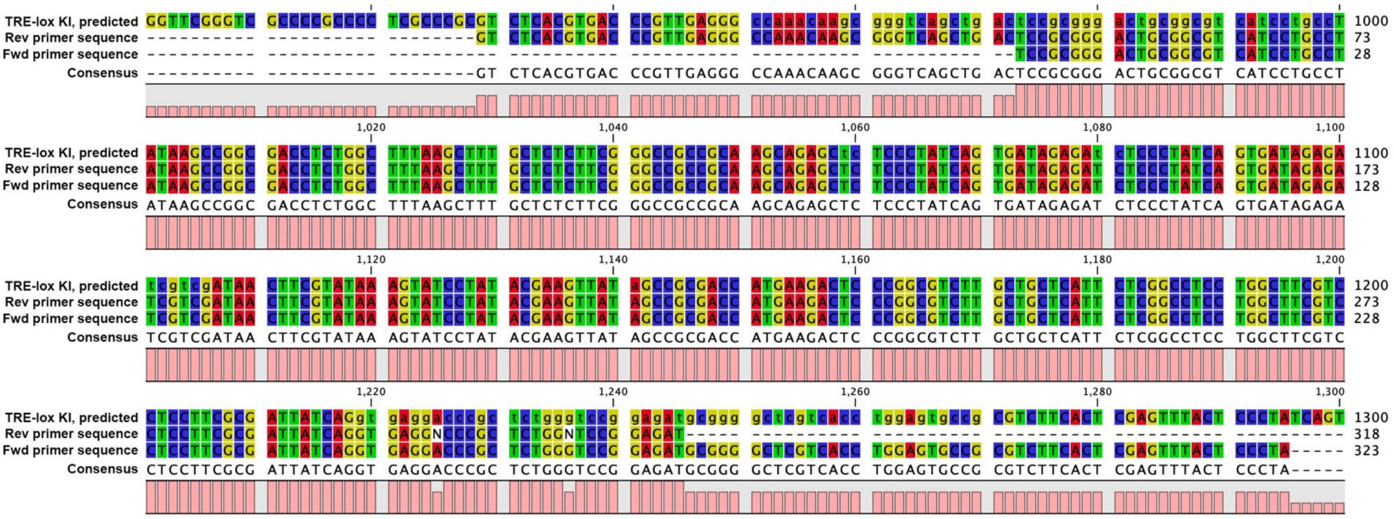

b. Alignment of sequencing of lower, NHEJ PCR amplicon from TL1C8 with WT sequence.

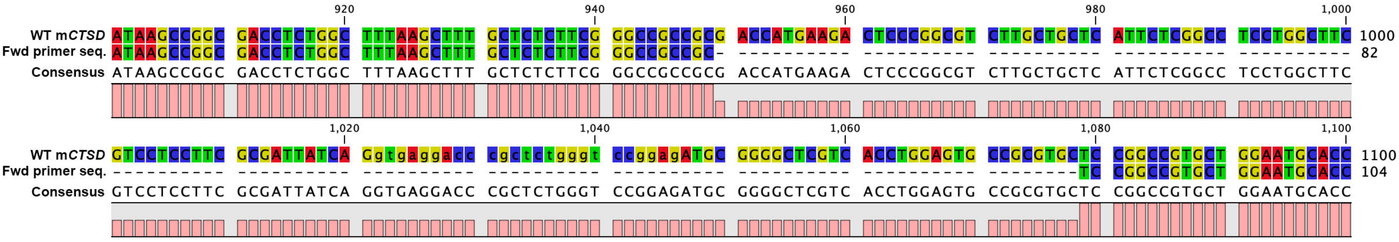

c. Alignment of sequencing of lower, NHEJ PCR amplicon from TL1C8 with predicted NHEJ sequence.

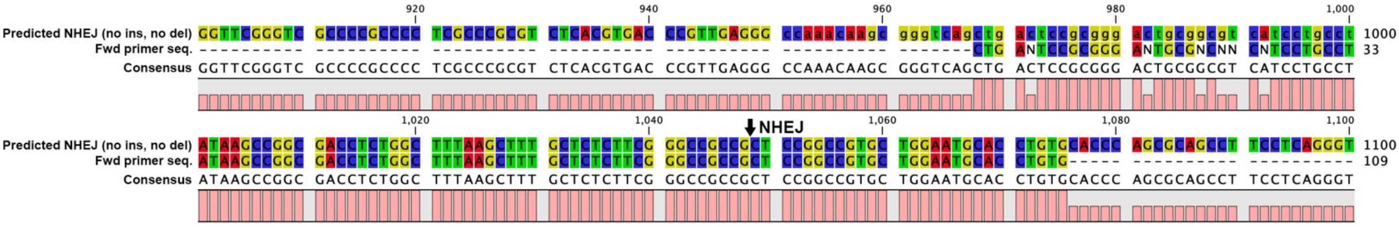

d. Sanger sequencing of lower, NHEJ PCR amplicon from TL1C8 stable clone.

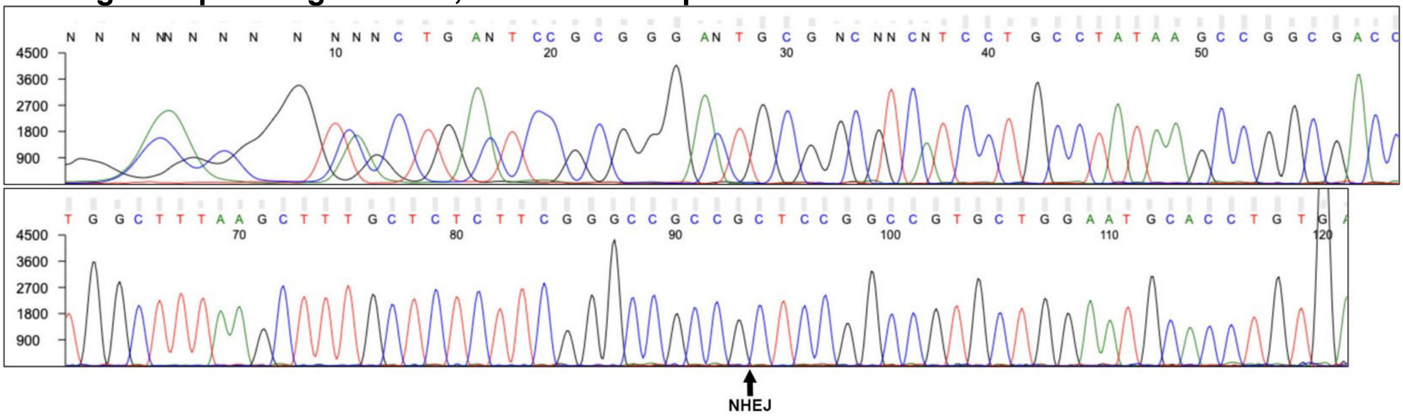

Supplementary Figure S6. PCR verification before vs. after Flp-mediated recombination.

a

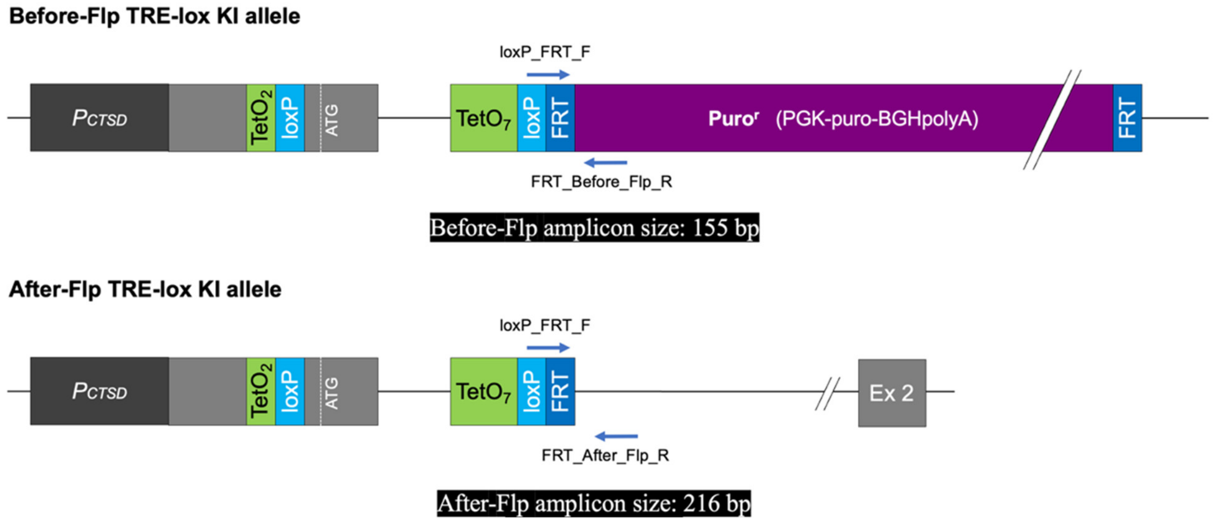

b

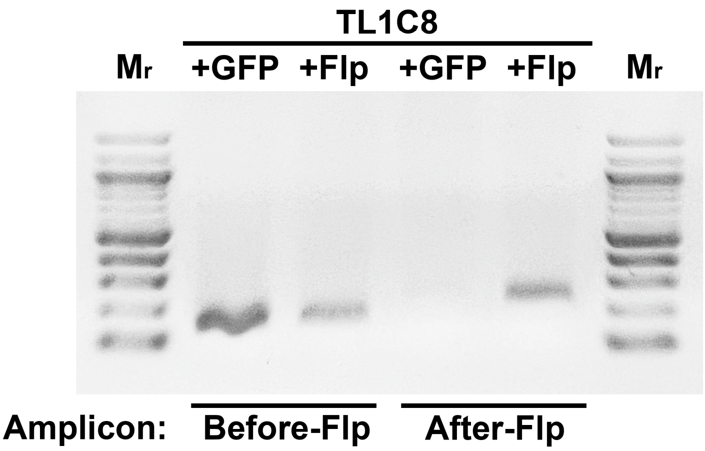

Note: The "After-Flp" amplicon is larger than the "Before-Flp" amplicon because the latter uses a different reverse primer located exclusively within the PuroR cassette.

Supplementary Figure S7. Dox dose-response curves for stable clones TFR1B11 and TFR2E9.

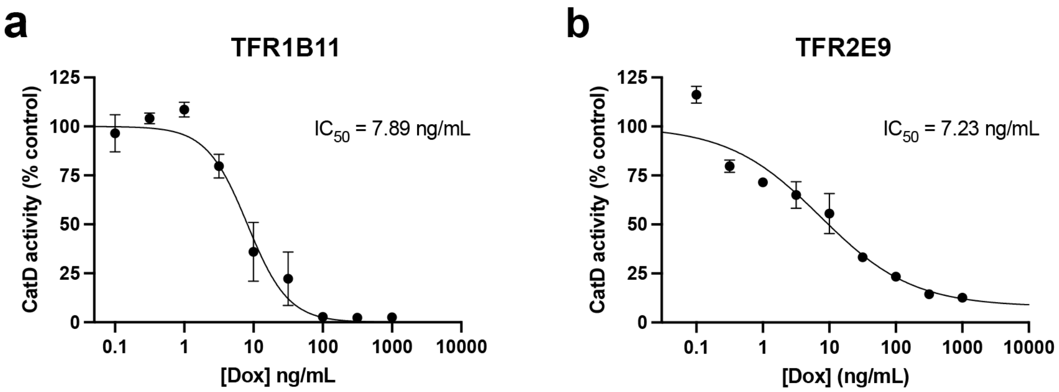

Supplementary Figure S8. Time courses of CatD downregulation in clones TFR1B11 and TFR2E9 after addition of Dox (100 ng/mL).

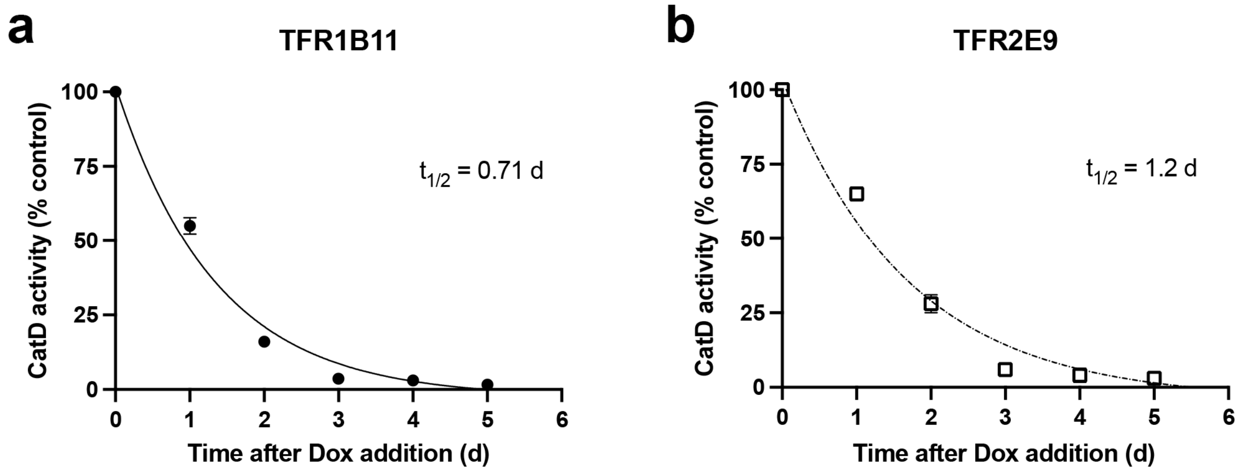

Supplementary Figure S9. Effect of rtTR on CatD activity in the presence and absence of Dox (1000 ng/mL) in transient pools and stable clones of TL1C8-Flp cells.

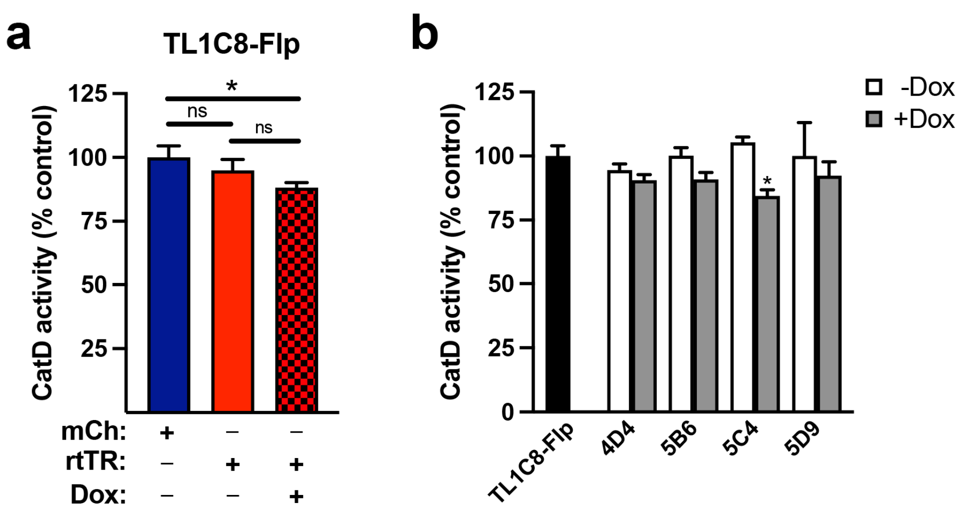

Supplementary Figure S10. Steady-state CatD protein levels in MEFs vs. TFR1D7 cells and in response to Dox addition and withdrawal, using CatD-KO line C1C5 as a control.

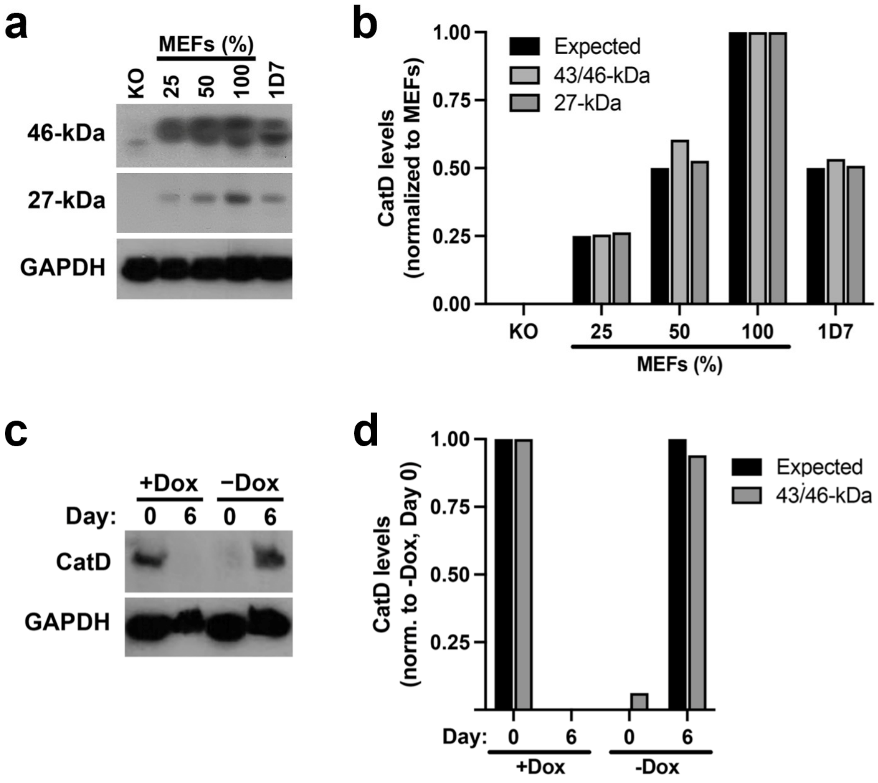

Table S1.   Primers used in this study.

Primer name	Sequence	Purpose
gRNA1_top	CACCGGGGAGTCTTCATGGTCGCGG	gRNA1 cloned into px330A-1x2
gRNA1_bottom	AAACCCGCGACCATGAAGACTCCCC	"
gRNA2_top	CACCGACCTGGAGTGCCGCGTGCTC	gRNA2 cloned into px330S-2, then px330A-1x2
gRNA2_bottom	AAACGAGCACGCGGCACTCCAGGTC	"
Vector_TetO2_F	GTCCAGTGTGGTGGAATTCAAGCAGAGCtCTCCCTATC	cloning of TetO2 for TRE-Lox KI template
TetO2_R	CGTATAGGATACTTTATACGAAGTTATCGACGATCTCTATCACTGAT	cloning of TetO2/LoxP sequence for KI template
mCTSD_loxP-Ex1_F	CGTATAAAGTATCCTATACGAAGTTATaGCCGCGACCATGAAGACTCC	cloning of LoxP/mCTSD region for KI template
mCTSD_3G_In1-2_R	GGAGTAAACTCGAGTGAAGACGCGGCACTCCAGGTGACGAG	cloning of mCTSD region/TRE3G for KI template
TRE3G_F	CGTCTTCACTCGAGTTTACTCC	cloning of TRE3G for KI template
TRE3G_R	CGACTAGTTATTACTACATAACTTCGTATAGGATACTTTATACGAAGTTAT<br>CTACCTCGACATACGTTCTC	cloning of TRE3G/LoxP for KI template
LoxP_FRT_F	CGAAGTTATGTAGTAATAACTAGTCGAAGTTCCTATTC	cloning of LoxP/FRT-flanked Puro^r for KI template/verification of Flp recombination
Vector_FRT_R	CCGCCACTGTGCTGGATATCAAATGGCGCGCCGAAGTTCC	cloning of FRT-flanked Puro^r for KI template
5'HA_mCTSD_TRE-Lox_KI_F	C*T*ATAAGCCGGCGACCTCTGGCTTTAAGCTTTGCTCTCTTCGGGCCGC<br>CGCAAGCAGAGCTCTCCCTATCAGTG	PCR amplifying TRE-Lox KI insert from template (asterisks denote phosphorothioate bonds)
3'HA_mCTSD_TRE-Lox_KI_R	G*A*CCCTGAGGAAGGCTGCGCTGGGTGCACAGGTGCATTCCAGCACG<br>GCCGAAATGGCGCGCCGAAGTTCC	"
mCTSD_WT_prom_F	GCGTCATCCTGCCTATAAGCCGGCG	genotyping of WT vs KI vs NHEJ mCTSD alleles/ verification of Cre recombination
mCTSD_WT_2R	GTCTCACGTGACCCGTTGAGG	"
mCTSD_TRE-Lox_2R	AACTCGAGTGAAGACGCGGCAC	"
FRT_Before_Flp_R	AGCGCATGCTCCAGACTGCC	verification of Flp recombination
FRT_After_Flp_R	CCTACTGTACCCTCCTGTTCG	"
mCTSD_KI_Cre_verification_R	CTTCGACTAGTTATTACTACATAACTTCG	verification of Cre recombination
pICN_KOZ_rtTR(V16)_F	CTTAATACGACTCACTATAGGCTAGCCGCCACCATGTCTAGACTGGACA<br>AGAGC	cloning of rtTR(V9I)/KRAB into pICherryNeo
KRAB_rtTR(V16)_R	CGAGCCGCTTTCGCACTTTAGCTG	"
rtTR(V16)_KRAB_F	CAGCTAAAGTGCGAAAGCGGCTCGCCAAAAAAGAAGAGAAAGGTCG	"
IRES_KRAB_only_R	CTCTAGAGGTACCACGCGTGAATTACACCAGCCAGGGCTCTTCTCC	"
DEL_KRAB_69oC_F	TAATTCACGCGTGGTACCTCTAGAG	deletion of KRAB from rtTRKRAB in pICherryNeo
DEL_KRAB_69oC_R	GCCGCTTTCGCACTTTAGCTGTTTC	"
